# Pathogen infection alters the gene expression landscape of transposable elements in *Drosophila melanogaster*

**DOI:** 10.1093/g3journal/jkae171

**Published:** 2024-08-12

**Authors:** Sabrina L Mostoufi, Nadia D Singh

**Affiliations:** Department of Biology, Institute of Ecology and Evolution, University of Oregon, Eugene, OR 97403, USA; Department of Biology, Institute of Ecology and Evolution, University of Oregon, Eugene, OR 97403, USA

**Keywords:** infection, transposable elements, bacteria, virus, fungi, Wolbachia, *Drosophila melanogaster*

## Abstract

Transposable elements make up substantial proportions of eukaryotic genomes and many are thought to be remnants of ancient viral infections. Current research has begun to highlight the role transposable elements can play in the immune system response to infections. However, most of our knowledge about transposable element expression during infection is limited by the specific host and pathogen factors from each study, making it difficult to compare studies and develop broader patterns regarding the role of transposable elements during infection. Here, we use the tools and resources available in the model, *Drosophila melanogaster*, to analyze multiple gene expression datasets of flies subject to bacterial, fungal, and viral infections. We analyzed differences in pathogen species, host genotype, host tissue, and sex to understand how these factors impact transposable element expression during infection. Our results highlight both shared and unique transposable element expression patterns between pathogens and suggest a larger effect of pathogen factors over host factors for influencing transposable element expression.

## Introduction

Transposable elements (TEs), or transposons, were first discovered by Barbara McClintock as genetic elements capable of moving around the genome and affecting kernel coloration in *Zea mays* (McClintock 1950). Many TEs are believed to have originated from ancient viral DNA that became integrated into a host's genome during a viral infection. TEs make up anywhere from 5% to 90% of eukaryotic genomes (Guio and González 2019). Though the field originally adhered to the belief that TEs were selfish elements that needed to be transcriptionally silenced, we are now beginning to understand the crucial roles TEs play in genome evolution, gene regulation, and host development for almost all eukaryotes (for review, see Cosby *et al*. 2019).

Today, TEs are organized via several levels of classification. The highest level is class and relates to the method of TE transposition. The most common classes are Class I or RNA transposons, which use reverse transcriptase to replicate throughout a host genome via an RNA intermediate, and Class II or DNA transposons, which include TEs that use transposase to invade host genomes. Within classes, TEs are further classified into superfamilies based on sequence similarity and evolutionary history. Finally, at the family level, different TEs vary in their copy number and locations across host genomes.

There are several potential fates for TEs, including: (1) hosts develop ways to suppress disruptive TEs or (2) TEs mutate and adaptation enables the TE to become an integral part of a host's gene expression networks (for review, see Cosby *et al.* 2019). TEs that still retain the ability to transpose are generally considered disruptive for their potential to insert themselves into gene regions and interrupt proper gene expression. To combat the negative effects associated with these disruptive TEs, hosts have developed methods for silencing their expression or preventing their transposition. One of the most widespread methods is small RNA (sRNA)-mediated silencing of TEs. An example of an sRNA method is the piRNA pathway in *Drosophila melanogaster*, which uses degraded copies of TEs to form piRNA clusters that regulate TE expression in the germline (Kelleher *et al*. 2020). Additionally, ectopic recombination between homologous TEs can result in negative selection against TEs with high copy numbers, long sequences, or which transpose into highly active regions of the genome (Petrov *et al.* 2003, 2011; Kelleher *et al*. 2020). Due to these mechanisms of TE control, many TEs in *D. melanogaster* are found in areas with low recombination and few genes.

However, not all TE insertions are harmful. Several cases have been documented where insertions of TEs disrupt gene functions in surprising ways and create new and beneficial phenotypes (for review, see González and Petrov 2009). In one example, the insertion of the TE *Doc1420* into the gene *CHKov1* created a new, truncated allele that confers pesticide resistance in *D. melanogaster* (Aminetzach *et al*. 2005). Others have shown that TE insertions into the gene *hsp70Ba* promoter are associated with changes in thermotolerance and female reproductive success in some *D. melanogaster* populations (Michalak *et al.* 2001; Lerman et al. 2003; Lerman and Feder 2005). There are also several TEs which have a high population frequency and display evidence for contributing to adaptation of *D. melanogaster* populations spreading out of Africa (González *et al.* 2008).

Additionally, some TEs have instead evolved to become integrated into host gene networks. In many cases, TEs have taken on the role of promoter, enhancer, or insulator and impact the expression of nearby genes. As an example, in *D. melanogaster*, TEs from the *Ty3* (*gypsy*) superfamily can act as promoters or insulators for various genes depending on the genomic location of the TE insertion (Moschetti *et al*. 2020). TEs can also impact gene expression via changes in methylation, as seen in *Arabidopsis thaliana* (Stuart *et al*. 2016). TEs can also take on other roles in cellular processes beyond promoters. A well-known example of this phenomenon in *D. melanogaster* is the elements *TART* and *HeT-A*, which are major components of the telomere elongation system in flies (Moschetti *et al*. 2020).

These systems of regulation and suppression of TEs can become disrupted when the host experiences novel environmental conditions. As early as 1984, McClintock hypothesized that TE activation could occur in response to genome challenges (McClintock 1984). Evidence to support this hypothesis can be found in several systems. For example, in *Caenorhabditis elegans*, heat shock and aging can increase the expression of some TEs (Kurhanewicz *et al*. 2020; Li *et al*. 2021). Temperature stress, including both heat shock and low temperature exposure, impacts TE expression in both *Drosophila simulans* and *D. melanogaster* (Ratner *et al*. 1992; Giraud and Capy 1996; Vieira and Biémont 1996; Vasilyeva *et al*. 1999). Nutrient deficiencies can also lead to TE activation in *Escherichia coli* (Hall 1999) and in the wheat pathogen *Zymoseptoria tritici* (Fouché *et al*. 2020).

The effect of infection on TE expression is particularly interesting, given the nature of how TEs became integrated into host genomes initially. Prior to discovering the roles TEs play in host gene networks, many in the field initially theorized that hosts repressed all TEs, but that infection could reawaken those elements. This can be true in some cases, but the hypothesized mechanism is via deregulation, rather than reactivation. For example, in both *Drosophila* and *Rattus* species, the piRNA regulatory pathway can become saturated with pathogenic RNA during a viral infection, resulting in de-repression of native TEs (Durdevic *et al.* 2013; van Gestel *et al*. 2014; Roy *et al*. 2020). However, not every pathogen, host, or TE react the same, and the field has more recently seen TEs play a crucial role in immune system function during pathogen infection. In this case, TEs are upregulated early in viral infection as part of the immune response in both humans and mice (Macchietto *et al*. 2020), as well as in *Drosophila* (Tassetto *et al*. 2017; Roy *et al*. 2020).

Despite the many ways TEs are affected by and can affect the response of their host to infections, efforts to understand these interactions have yet to capture the broader dynamics of the system. Although many studies have investigated the response of TEs to infection, these studies have generally been limited to one host and one infection, and sometimes even a limited number of specific TEs. In particular, studies of TEs and viral infections are numerous (Magwire *et al.* 2011; Liu *et al.* 2018; Harsh *et al.* 2020; Macchietto *et al*. 2020; Roy *et al.* 2020; Lindsey *et al.* 2021; Hale 2022), but investigations using other types of infections are less common by comparison. Studies also often analyze different host species, genotypes, sexes, or tissue samples, as well as different pathogen species or strains. These differences make it difficult to understand the broader patterns of TE activation outside of a few, very specific circumstances, even in model organisms. One potential solution to this problem would be to conduct a large-scale study which directly measures the changes in TE expression within a single model organism while altering the type of infection (e.g. viral vs bacterial), host genotypes, and other factors of interest. However, the time, expense, skills, and facilities required for such a study can present significant barriers. An alternative, and more feasible, solution would be to compare numerous studies using the same model organism, allowing us to begin untangling the impact of these host and pathogen factors on TE expression. The fruit fly, *D. melanogaster*, is an ideal candidate for investigating these dynamics because of the number of tools available for TE annotation and the number of gene expression datasets available.

Here, we investigate broad-scale patterns in TE expression during infection of the model organism, *D. melanogaster*. We gathered RNA-seq samples from published datasets of *D. melanogaster* infected with a broad range of bacterial, fungal, and viral pathogens. We measured TE expression between control and infected samples and compared patterns both within and between pathogen groups to assess the effect of pathogen species, host genotype, host tissue sample, and host sex on TE expression. Our results show that the type of pathogen has a much larger effect on TE expression changes when compared to host factors. We also find that bacterial infections differ significantly from other types of infections, but that infections with the bacterium, *Wolbachia pipientis*, look more similar to fungal infections. These findings provide critical insight into how host and pathogen factors can impact TE activity during infection in *D. melanogaster*.

## Materials and methods

### Sequence processing

The datasets used in this study were downloaded from the European Nucleotide Archive (ENA) via the European Molecular Biology Laboratory's European Bioinformatics Institute website. Processing and analysis of RNA-seq files were completed using the University of Oregon's high performance computing cluster, Talapas. We used the program STAR (v2.7.9a) to align and merge paired fastq files using the *D. melanogaster* reference genome (Release 6.41), generating unsorted BAM (Binary Alignment Map) files for each sample (Dobin *et al*. 2013). To optimize the data for TE analysis, we used the recommended settings for STAR from Jin and Hammell (2018) by setting –outFilterMultimapNmax 100 and –winAnchorMultimapNmax 200, with default values for all other parameters.

### Measuring TE expression

We used the program TEtranscripts to count TE families in our selected datasets (Jin *et al*. 2015). TEtranscripts requires two GTF (General Transfer Format) files, one for gene sequences and one for TE sequences. We used the *D. melanogaster* TE GTF file (dm6_BDGP_rmsk_TE.gtf.gz) provided on the Hammell lab website (https://labshare.cshl.edu/shares/mhammelllab/www–data/TEtranscripts/TE_GTF/) and the *D. melanogaster* genome Release 6.32 GTF file (Drosophila_melanogaster.BDGP6.32.104.gtf.gz) from Ensembl (ftp.ensembl.org/pub/release-107/gtf/drosophila_melanogaster/). After alignment, BAM files for control and infection groups from each dataset were analyzed using TEtranscripts, with –mode multi and –norm TC and default parameters, to produce TE transcript counts and R Scripts for DESeq2 analysis. We used FlyBase (release FB2024_03) to find information on TEs identified in our analyses (Öztürk-Çolak *et al*. 2024).

When presenting our findings, we have also elected not to use the antiquated and problematic name for the *gypsy* TE family, instead opting for the alternate naming of *Ty3* as advocated for by others in the field (Wei *et al*. 2022).

### Statistical analyses

All statistical analyses and data visualization were completed in RStudio (2023.06.0, “Mountain Hydrangea” Release).

To evaluate whether TE expression changed during infection, we calculated the log2-fold change between control and infection samples for each dataset using the DESeq2 program (v1.32.0) (Love *et al*. 2014). DESeq2 assesses significance by measuring the fit of each gene to a generalized linear model and producing *P*-values adjusted for multiple testing; we identified TEs with an adjusted *P*-value less than or equal to 0.05 as differentially expressed TEs (DETEs). Each DETE, therefore, represents a TE family (e.g. *Invader4*) which showed a significant change in expression during infection within the analyzed dataset (e.g. Elya *et al*. 2018).

We ran several linear models, both to evaluate whether sample size or read counts affected the number of DETEs detected for each dataset and to analyze the overall effect size of each variable. To evaluate the effect of sample size on the number of DETEs detected in each dataset, we ran the following model: lm(DETEs ∼ number of samples per dataset). To evaluate the effect of read count on the number of DETEs detected in each dataset, we ran the same model as before, but with read count as the independent variable. To analyze the relative effect of each variable on the log fold change of DETEs, we ran the following model: glm(formula) = log2FoldChange ∼ pathogen + tissue + genotype + sex + 0, family = gaussian(link = “identity”).

We used a *χ*^2^ goodness of fit test to examine whether observed patterns of DETEs at class and superfamily levels were significantly different from proportions of TEs present in the *D. melanogaster* genome, as well as to compare differences between and within pathogen groups. Genomic proportions of TEs categorized by class and superfamily were calculated from the *D. melanogaster* genome Release 6.32 Ensembl GTF annotation file.

## Results

In this study, we analyzed changes in TE expression between control and infected *D. melanogaster* using RNA-seq data from 14 published datasets (Table 1). Together, these datasets include 30 unique fly genotypes and 19 species of single and multi-species infections of bacterial, fungal, and viral pathogens, with some non-pathogenic bacterial infections also included. We identified DETEs families by identifying TEs with significantly increased or decreased expression when comparing infection and control treatments within the analyzed datasets (see “Methods” for detailed description).

**Table 1.** The RNA-Seq datasets used in this study.

Paper (BioProject)	Pathogen group	Species/strain	Control	Infection	Genotypes	Tissue	Sex
Dobson *et al*. (2016) (PRJNA347655)	Bacteria	*Acetobacter pomorum* (*DmCS_004*) and *A. tropicalis* (*DmCS_006*) and *Lactobacillus brevis* (*DmCS_003*) and *L. fructivorans* (*DmCS_002*) and *L. plantarum* (*DmCS_001*)	GF	MS	*B04*; *B10*; *B17*; *Canton S*; *I16*; *I34*; *I38*; *N01*; *N04*; *N15*; *RAL306*; *RAL318*; *RAL42*; *RAL555*; *T01*; *T05*; *T29*; *ZW184*	Whole body	Male
Guo *et al*. (2014) (PRJNA232924)	Bacteria	Conventional microbiome	GF	CV	*OreR*	Intestine	Female
Troha *et al*. (2018) (PRJNA428174)	Bacteria	*Enterococcus faecalis*, *Erwinia carotovora* (*Ecc15*), *E. coli*, *Micrococcus luteus*, *Providencia rettgeri*, *P. sneebia*, *Pseudomonas entomophila*, *Serratia marcescens* (*Db11*), *S. marcescens* (*Type*), *Staphylococcus aureus*	CV	SS	*CantonS*	Whole body	Male
Elya *et al*. (2018) (PRJNA435715)	Fungi	*Entomophthora muscae*	CV	SS	*CantonS*	Brain, carcass	Female
Moskalev *et al*. (2015) (PRJNA295562)	Fungi	*Beauvaria bassiana*	CV	SS	*Canton S*	Whole body	Male
Paparazzo *et al*. (2015) (PRJNA279177)	Fungi	*Beauvaria bassiana*	CV	SS	*Africa*, *America*, *Asia*, *Europe*	Whole body	Male
Ramírez-Camejo and Bayman (2020) (PRJNA377735)	Fungi	*Aspergillus flavus*	CV	SS	*Canton S*	Whole body	Female
Harsh *et al*. (2020) (PRJNA533975)	Virus	*ZIKV* (*MR766*)	CV	SS	*w1118*	Whole body	Female
Roy *et al*. (2020) (PRJNA540249)	Virus	*SINV*	CV	SS	*w1118*	Carcass, ovary	Female
Lindsey *et al*. (2021) (PRJNA682591)	Virus, Wolbachia	*SINV*, *W. pipientis* (*wMel2*)	CV	SS, MS	*w1118*	Whole body	Female
Detcharoen *et al*. (2021) (PRJNA602188)	Wolbachia	*W. pipientis* (wMel)	CV	SS	*w1118*	Whole body	Female
Frantz *et al*. (2023) (PRJNA936147)	Wolbachia	*W. pipientis*	CV	SS	*RAL73*, *RAL306*, *RAL783*, *RAL853*	Ovary	Female
Grobler *et al*. (2018) (PRJNA483452)	Wolbachia	*W. pipientis*	CV	SS	*JW18*	Cell culture	Unknown
He *et al*. (2019) (PRJNA439370)	Wolbachia	*W. pipientis*	CV	SS	Unknown	Ovary	Female

For each dataset, information on the pathogen group (bacteria, fungi, virus, or Wolbachia), the specific species or strain of pathogen, the “control” and “infection” treatments of each study, the host genotypes included, the sample tissue, and sex of flies are described. In datasets where the specific strain is known for a given pathogen species, the strain is specified in parentheses after the species name; for pathogens where no strain was identified in the original paper, only the species name is included in this table. Flies with a conventional microbiome are represented by “CV”, germ-free flies are represented by “GF”, single-species infections are represented by “SS”, and multi-species infections are represented by “MS”. All samples, with the exception of the cell culture samples, came from adult flies.

**Fig. 1.**
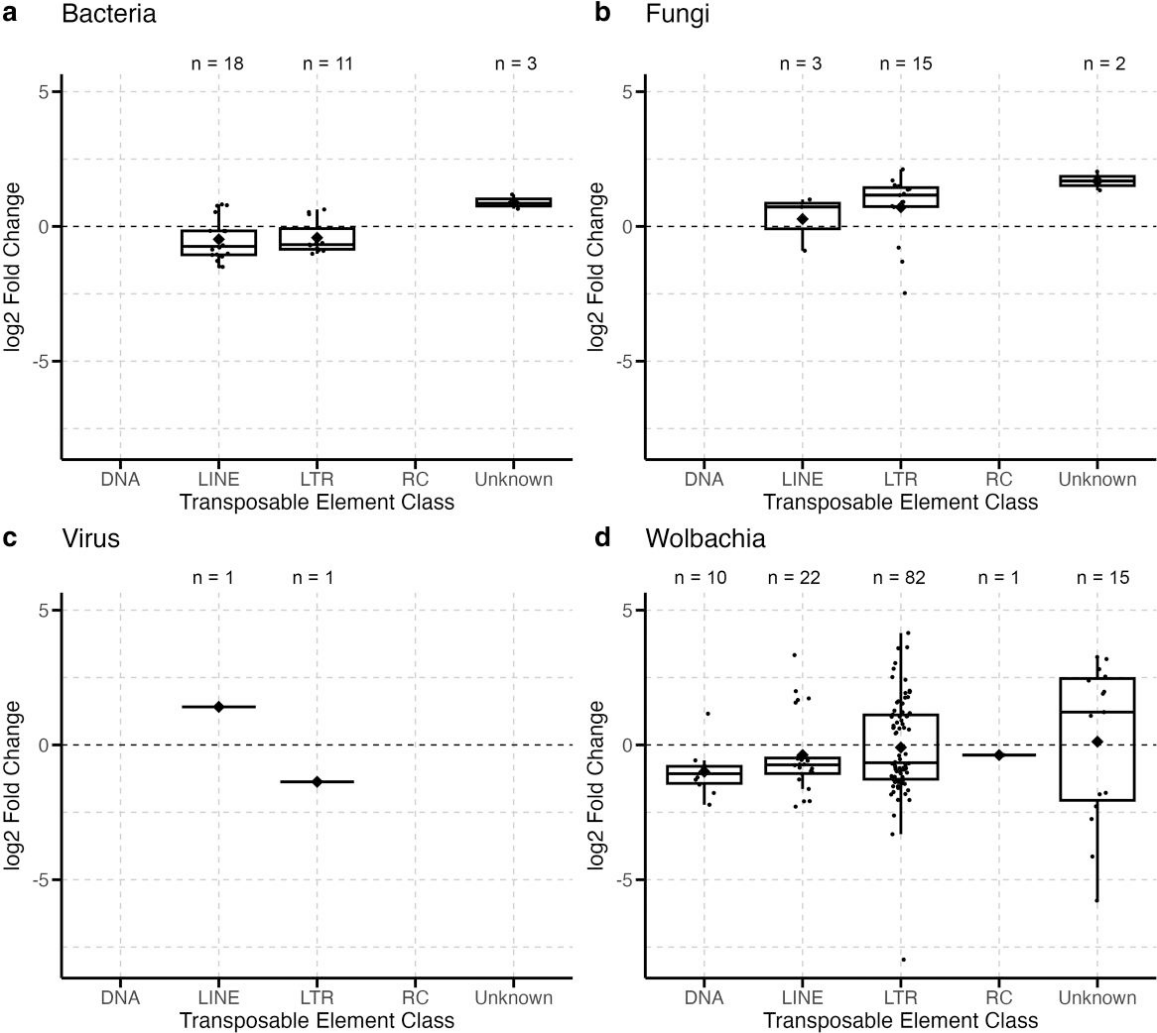
Different infections significantly impact classes of TEs expressed during infection in *D. melanogaster*. Each point represents a TE family with significant differential expression during infection by either bacteria (a), fungi (b), virus (c), or *Wolbachia* (d) within one of the analyzed datasets. Boxplots present summary statistics, where the top and bottom edges encompass the first to third quartiles and the middle bar represents the median for each group. Boxplot whiskers extend to the smallest and largest nonoutliers. The black diamond in each box represents the average TE expression fold change for each data group. The number of DETEs in each boxplot is denoted by “*n*” at the top of each graph. TE classes are: DNA, LINEs, LTRs, RC, and unknown.

Our analyses revealed 184 DETEs affected by infection in flies, which included 115 unique TE families, 15 superfamilies, and 5 classes. The full list of DETEs is available in Supplementary Table 1. Of all DETEs, 20 came from fungal infection datasets, 2 came from viral infection datasets, and 162 came from bacterial infection datasets, of which 130 were from *W. pipientis* infections and 32 from non-*Wolbachia* infections (Fig. 1). Due to these differences in DETEs among the datasets, we tested whether there was a correlation between sample size, read count, and the number of significant TEs within each dataset using linear models. We found no significant correlation between dataset sample size and the number of significant TEs (*R*^2^ = −0.0016, *P*-value = 0.34) or between dataset read counts and the number of significant TEs (*R*^2^ = −0.07, *P*-value = 0.75).

To investigate general patterns in expression across the variables present in our study, we used a generalized linear model to evaluate the effect of pathogen, host genotype, host tissue, and host sex on the change in expression of DETEs during infection. Results from this model showed marginally nonsignificant results for different pathogens, tissue types, and host genotypes, suggesting that these factors did not have large, homogenous effects on expression changes in DETEs. This aligned with our expectations based on visualizations of the data, which showed no consistent patterns in whether DETEs had increased or decreased expression during infection for different variables, as seen for pathogen type (Fig. 1). Instead, we observed that different variables seemed to affect the types of TEs affected, rather than the magnitude of expression, which is why we focused our analyses on patterns of TE class and superfamily in the following sections.

### Comparisons of infection by different pathogens

To understand how infection affects TE activity, we compared TE expression between flies infected with different pathogens, including bacterial, fungal, and viral infections. We analyzed DETEs at the level of class and superfamily classification and the change in expression, whether increased or decreased, in order to identify patterns in TE activity. Due to the large number of datasets with *W. pipientis*, and because the native strain of *Wolbachia*, *wMel*, is generally not considered pathogenic in *D. melanogaster* (Fry *et al.* 2004), we considered *Wolbachia* infection separately from other bacterial infections. Due to the small number of DETEs identified in viral infection datasets, we were unable to include them in the following statistical tests for pathogen-level analyses.

First, we tested whether there were significant differences in patterns of TE expression between different types of pathogens. Bacterial infections significantly affected different classes and superfamilies of TEs compared to all other infection types (Table 2). Fungal and *Wolbachia* infections affected similar proportions of TE classes and superfamilies, and there were no significant differences among these groups (Table 2). Pathogen groups also significantly differed in the proportion of TEs with increased or decreased expression during infection (*c*^2^ = 13.79, df = 3, *P*-value = 3.2e−3). Bacterial and *Wolbachia* infections caused 62% of DETEs in those datasets (*n* = 101) to decrease in expression while 80% of DETEs (*n* = 16) in fungal infection datasets increased in expression during infection.

**Table 2.** Results from *χ*^2^ comparisons of class and superfamily TE proportions between pathogen groups.

		Class		Superfamily	
		Fungi	*Wolbachia*	Fungi	*Wolbachia*
Bacteria	*c* ^2^	7.52	22.59	13.48	25.96
	df	1	4	5	14
	*P*-value	6.1e−3**	1.5e−4***	0.019*	0.026*
Fungi	*c* ^2^		2.15		4.71
	df		4		14
	*P*-value		0.71		0.99

The proportions of DETEs, classified by TE class or superfamily, were compared between each pathogen group. Each group in the table represents the results from a *χ*^2^ test between the pathogen groups in the associated row and column for either TE class or TE superfamily proportions. Asterisks indicate significance: * < 0.05, ** < 0.01, *** < 0.001.

**Fig. 2.**
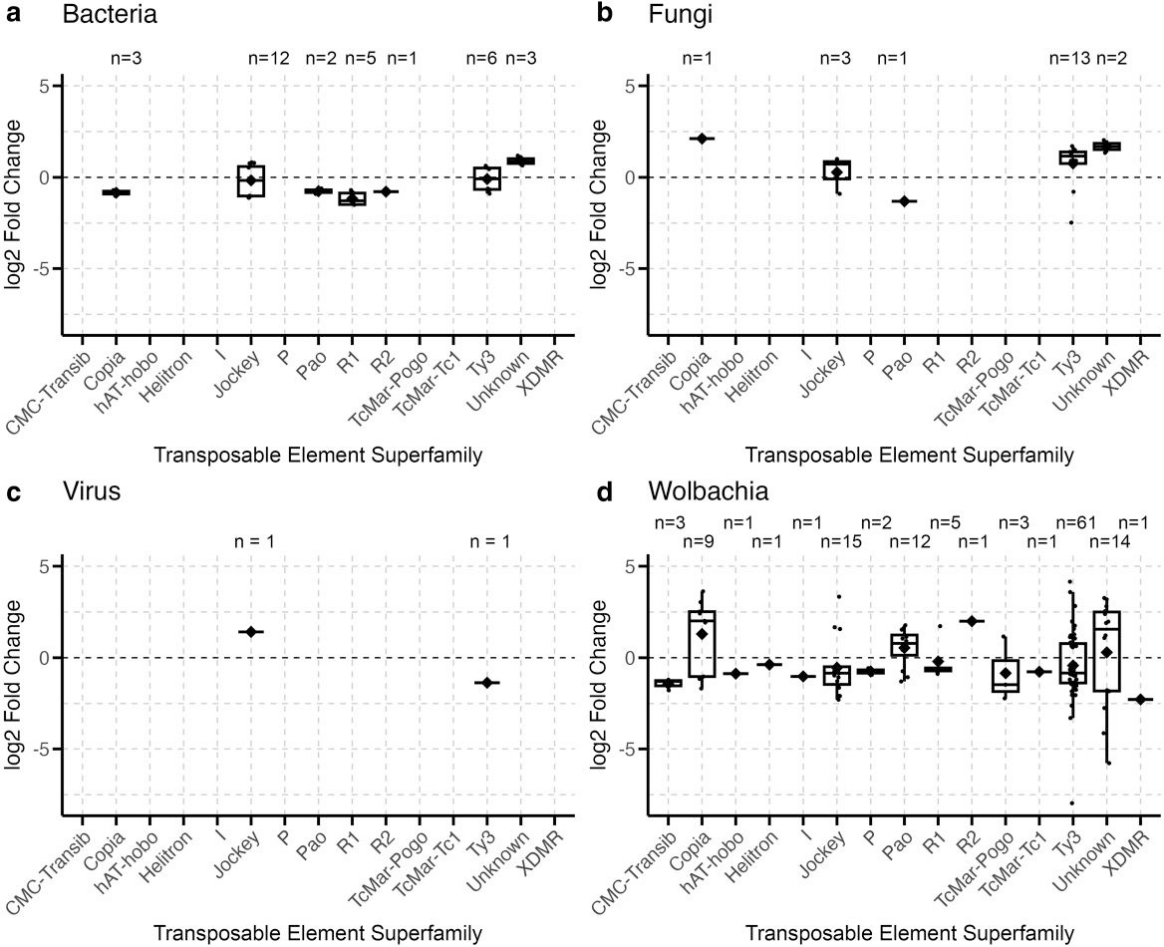
Different infections significantly impact superfamily identity of TEs expressed during infection in *D. melanogaster.* Each point represents a DETE family during infection by bacteria (a), fungi (b), virus (c), or *Wolbachia* (d) within one of the analyzed datasets. Boxplots present summary statistics, where the top and bottom edges encompass the first to third quartiles and the middle bar represents the median for each group. Boxplot whiskers extend to the smallest and largest nonoutliers. The black diamond in each box represents the average TE expression fold change for each data group. The number of DETEs in each boxplot is denoted by “*n*” at the top of each graph.

Fungal and *Wolbachia* infections affected TEs primarily from the long terminal repeat (LTR) class, with *Ty3* being the most abundant TE superfamily represented (Figs. 1 and 2). However, LTR elements make up approximately 50% of TEs in the *D. melanogaster* genome, 35% of which are from the *Ty3* superfamily. This raised the question of whether these types of infections specifically affected LTR and *Ty3* TEs, or whether their expression was a result of broad and nonspecific activation of TEs during infection. Therefore, we tested whether the class and superfamily proportions of TEs differentially expressed during each infection differed from proportions of TEs present in the *D. melanogaster* genome. These analyses are presented below.

### Effect of fungal infections on TE expression

Our analyses of fungal infections included four datasets of single-species infections, including *Aspergillus flavus*, *Beauvaria bassiana*, and *Entomophthora muscae* (Table 1). Comparison of control and infected samples resulted in 20 DETEs. Fungal infections showed a bias for increasing expression of DETEs, where 80% (*n* = 16) of all DETEs increased in expression during infection. Of the 20 DETEs, 75% (*n* = 15) belonged to the LTR class (Fig. 1b), and 65% (*n* = 13) came from the *Ty3* superfamily (Fig. 2b).

We also evaluated whether fungal infections broadly affected TEs relative to genomic proportions, or if certain classes or superfamilies of TEs specifically respond to fungal infection. To test this, we conducted a *χ*^2^ goodness of fit test to compare the proportions of DETE class and superfamily identity with the genomic proportions of TE classes and superfamilies in the *D. melanogaster* genome. At the class level, DETEs were significantly different from the genomic proportions of TE classes (*c*^2^ = 19.58, df = 8, *P*-value = 0.012), specifically that there were more unknown DETEs than expected (*χ*^2^, residual = 3.12849237). However, at the superfamily level, DETEs did not differ significantly from expected proportions (*c*^2^ = 15.98, df = 30, *P*-value = 0.98).

### Effect of viral infections on TE expression

To assess the effect of viral infections on TE expression, we analyzed three datasets which included infection with the Zika virus (ZIKV) and Sindbis virus (SINV) (Table 1). Across the datasets, there were only 2 DETEs, one each from the long interspersed nuclear element (LINE) and LTR classes (Fig. 1c) and belonging to the *Jockey* and *Ty3* superfamilies (Fig. 2c).

### Effect of bacterial infections on TE expression

We analyzed one dataset comparing infection with 10 different bacterial species in *D. melanogaster*, and two datasets with conventional and germ-free flies (Table 1). With respect to the former, infection with novel pathogenic and non-pathogenic bacterial species resulted in 31 DETEs, 71% (*n* = 22) of which displayed decreased expression during infection. Over 50% (*n* = 17) of DETEs belonged to the LINE class (Fig. 1a), and the most common superfamily was *Jockey*, representing 39% (*n* = 12) of DETEs (Fig. 2a). With respect to comparisons between conventional and germ-free flies, there was only one DETE.

The class proportions of the 31 DETEs in the novel bacterial infection datasets differed significantly from expected proportions based on the *D. melanogaster* genome (*c*^2^ = 45.41, df = 8, *P*-value = 3.08e−07), with more TEs from the LINE (*χ*^2^, residual = 4.57) and unknown classes (*χ*^2^, residual = 3.67) and fewer TEs from the rolling circle (RC) class (*χ*^2^, residual = −2.15) compared to TE class proportions in the genome. Superfamily proportions also differed significantly from expected proportions (*c*^2^ = 90.70, df = 30, *P*-value = 5.14e−08), with more TEs from the *Copia* (*χ*^2^, residual = 2.59), *Jockey* (*χ*^2^, residual = 5.02), *R*1 (*χ*^2^, residual = 3.91), and *R*2 superfamilies (*χ*^2^, residual = 5.51) and fewer TEs from the *Helitron* superfamily (*χ*^2^, residual = −2.04) than expected.

Within the 10 bacterial species, there were 7 Gram-negative and 3 Gram-positive species. We tested whether there were differences within this dataset based on Gram classification of bacterial species. We found no significant differences between Gram-negative and Gram-positive bacteria species for TE class (*c*^2^ = 5.60, df = 6, *P*-value = 0.47) or TE superfamily (*c*^2^ = 10.74, df = 16, *P*-value = 0.83). In addition, Gram-negative and Gram-positive bacteria had very similar proportions of TEs with increased or decreased expression, with approximately 70% of TEs decreasing in expression during infection.

### Effect of *Wolbachia* infections on TE expression

We analyzed five datasets of *Wolbachia* infection in *D. melanogaster*, including the *wMel* and *wMel2* variants and one dataset of co-infection between *Wolbachia* and SINV (Table 1). Across the datasets of *Wolbachia*-only infections, there were 130 DETEs, 63% (*n* = 82) of which belonged to the LTR class (Fig. 1d), and 47% (*n* = 61) of all DETEs were from the *Ty3* superfamily (Fig. 2d). Our analyses identified no DETEs in flies co-infected with *Wolbachia* and SINV.

Similar to other bacterial infections, *Wolbachia* infection caused a majority of TEs to decrease in expression, with approximately 61% (*n* = 79) of DETEs decreasing in expression during infection. We found that class proportions of DETEs were significantly different from the proportions of TEs found in the *D. melanogaster* genome (*c*^2^ = 122.05, df = 8, *P*-value < 2.2e−16), with more TEs from the LTR (*χ*^2^, residual = 2.64) and unknown classes (*χ*^2^, residual = 9.416), and less from the RC class (*χ*^2^, residual = −4.09) than expected. Superfamily proportions also differed from expected genome proportions (*c*^2^ = 98.59, df = 30, *P*-value = 3.10e−09), specifically that there were more *Copia* (*χ*^2^, residual = 3.45), *R*2 (*χ*^2^, residual = 2.49), *TcMar-Pogo* (*χ*^2^, residual = 5.90), and *Ty3* TEs (*χ*^2^, residual = 3.13) and less *CR*1 (*χ*^2^, residual = −2.11) and *Helitron* TEs (*χ*^2^, residual = −3.84) than expected.

### Comparison of host factors during infection

Across the datasets we analyzed, samples varied by host sex, genotype, and tissue. To assess the effect of these factors on TE expression during infection, we compared the expression of DETEs during infection across datasets of different host sex, host genotypes, and tissue samples. Similar to our analyses of pathogen type, we analyzed DETEs at the level of class and superfamily classification and the direction of changes in expression between different host factors during infection.

### Effect of host sex on TE expression during infection

**Fig. 3.**
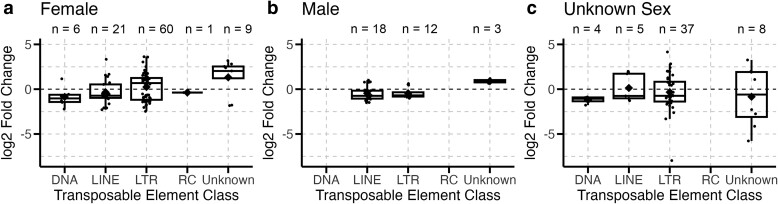
Host sex has a minimal effect on TE activity during infection. The TE families differentially expressed during infection are grouped by female samples (a), male samples (b), and a sample of unknown sex (c). Boxplots present summary statistics, where the top and bottom edges encompass the first to third quartiles and the middle bar represents the median for each group. Boxplot whiskers extend to the smallest and largest nonoutliers. The black diamond in each box represents the average TE expression fold change for each data group. The number of DETEs in each boxplot is denoted by “*n*” at the top of each graph.

First, we analyzed whether host sex had a significant effect on TE expression during infection. Our datasets included 10 datasets with samples from female flies, four datasets of male flies, and one dataset of *D. melanogaster* cell culture samples of an unspecified sex. Of the total DETEs analyzed in our study, female samples accounted for 97 DETEs, compared to 33 DETEs in male samples and 54 DETEs in samples of unknown sex (Fig. 3).

Approximately 52% (*n* = 50) of DETEs in female samples showed decreased expression during infection, compared to male and unknown sex samples in which 64% (*n* = 56) of DETEs decreased in expression during infection. Since the unknown sex samples came from a single infection dataset, we excluded this dataset when testing whether host sex had an effect on class and superfamily proportions of DETES. These differences in expression were not significantly different between samples of different host sexes (*c*^2^ = 32.60, df = 1, *P*-value = 0.11). Host sex significantly affected the proportions of the classes (*c*^2^ = 14.15, df = 4, *P*-value = 6.82e−3) but not the superfamilies (*c*^2^ = 18.07, df = 11, *P*-value = 0.08) of TEs that were differentially expressed during infection.

However, unequal associations between pathogen groups and host sex in our datasets may have caused the effect of sex to be confounded by the effect of pathogen. For example, female samples were more likely to have higher proportions of DETEs from the LTR class (*χ*^2^, residual = 2.54), likely because a majority of female samples came from *Wolbachia* infection datasets. Similarly, male samples had higher proportions of LINE DETEs (*χ*^2^, residual = 3.56) and were also primarily from non-*Wolbachia* bacterial infection datasets. To disentangle this association, we tested for the effect of sex in fungal infections, which had equal numbers of female and male fly datasets. We found no significant effect of sex on the class identity of DETEs (*c*^2^ = 2.22, df = 2, *P*-value = 0.33). However, female samples still had higher counts of DETEs than male samples, consisting of 18 DETEs vs 2 DETEs in the respective sexes.

### Effect of host genotype on TE expression during infection

**Fig. 4.**
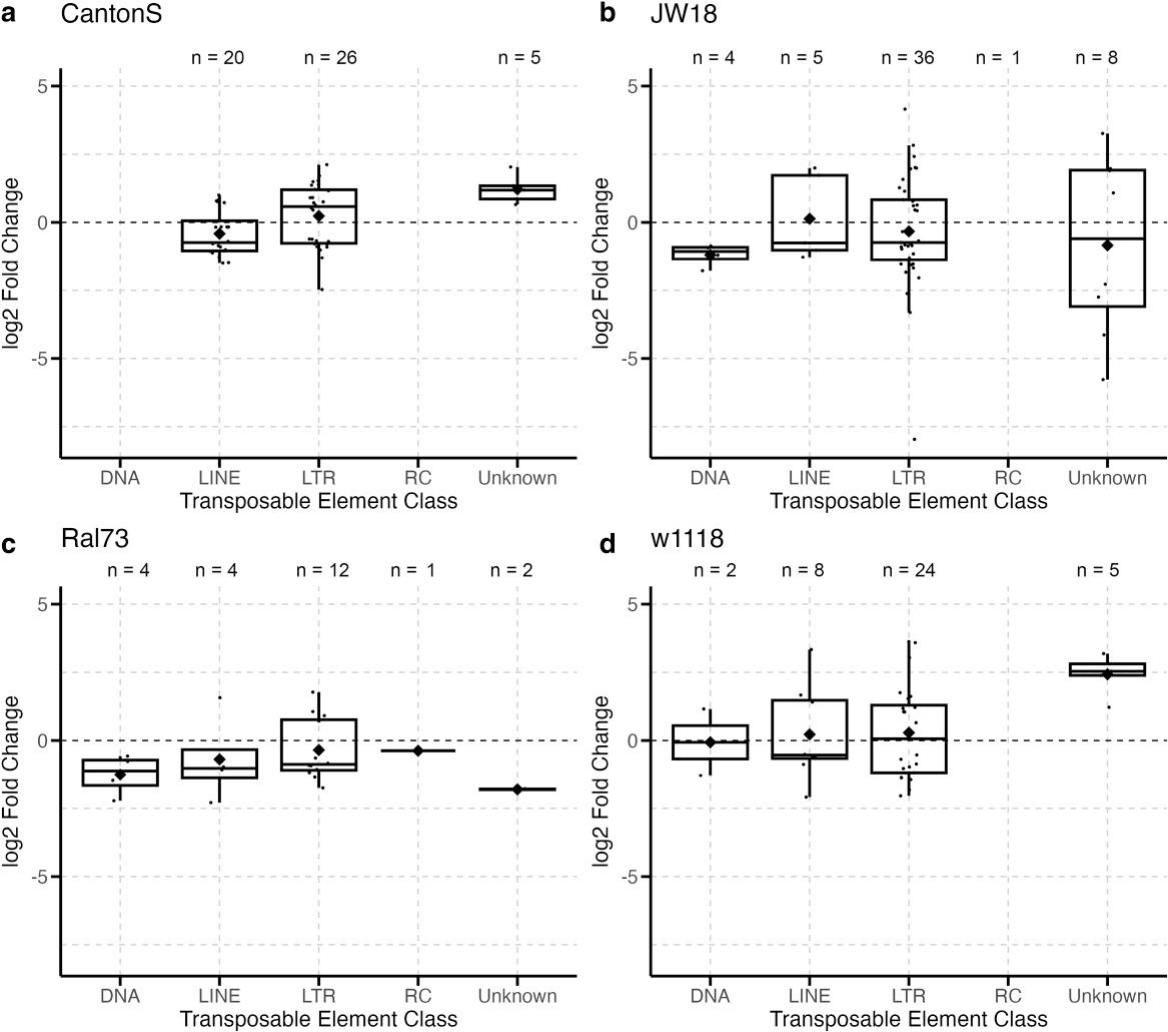
Host genotype has a minimal effect on differences in TE expression during infection. A total of 16 different host genotypes were analyzed for their effect on the classes of different TE families expressed during infection in *D. melanogaster*. Only a subset of genotypes with the most DETEs are presented in this figure. Each point represents a single TE family that was differentially expressed during infection in one of the analyzed datasets in either the *CantonS* (a), *JW18* (b), *Ral73* (c), or *w1118* (d) fly genotype. Boxplots present summary statistics, where the top and bottom edges encompass the first to third quartiles and the middle bar represents the median for each group. Boxplot whiskers extend to the smallest and largest nonoutliers. The black diamond in each box represents the average TE expression fold change for each data group. The number of DETEs in each boxplot is denoted by “*n*” at the top of each graph.

Additionally, the samples in our study included 30 different host genotypes (Table 1). However, due to the sample size of each genotype within the Dobson *et al.* (2016) dataset, we were unable to include it in this particular analysis, reducing our final analysis to 16 unique host genotypes. Of these genotypes, only 8 genotypes had DETEs during infection, the most prominent of which are presented in Fig. 4. Most genotypes displayed a bias toward decreased expression of DETEs during infection, ranging from 53% to 86% of the total DETEs for each genotype.

To test for the effect of host genotype on TEs expressed during infection, we used *χ*^2^ analyses to evaluate differences in expression and whether specific TE classes or superfamilies were differentially expressed in different host genotypes. There was no significant effect of host genotype on the direction of TE expression (*c*^2^ = 14.24, df = 8, *P*-value = 0.076), the class proportions (*c*^2^ = 41.68, df = 32, *P*-value = 0.12), or the superfamily proportions of DETEs (*c*^2^ = 78.95, df = 112, *P*-value = 0.99) expressed during infection.

### Effect of tissue sample on TE expression during infection

Finally, we also examined the effect of tissue sample on TE expression during infection. Our datasets included samples from six different tissues, including brain, carcass (whole body with ovaries removed), cell culture, intestines, ovaries, and whole body flies (Table 1).

**Fig. 5.**
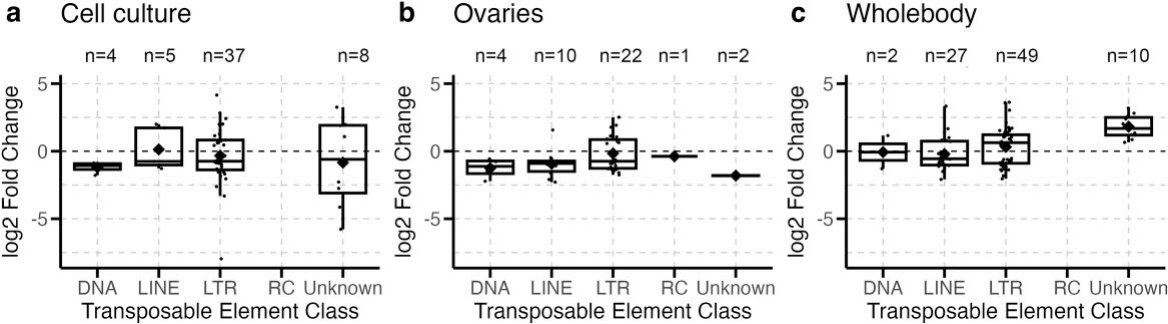
Ovarian samples display decreased TE expression during infection. Each point represents a single TE family that was differentially expressed during infection in cell culture (a), ovary (b), or whole body (c) fly tissue samples within one of the analyzed datasets. Boxplots present summary statistics, where the top and bottom edges encompass the first to third quartiles and the middle bar represents the median for each group. Boxplot whiskers extend to the smallest and largest nonoutliers. The black diamond in each box represents the average TE expression fold change for each data group. The number of DETEs in each boxplot is denoted by “*n*” at the top of each graph.

We tested whether DETEs varied significantly by host tissue and found that tissue did not significantly affect the class proportions (*c*^2^ = 21.08, df = 16, *P*-value = 0.18) or superfamily proportions of TEs (*c*^2^ = 45.27, df = 56, *P*-value = 0.85) (Fig. 5). Host tissue significantly affected the direction of expression of DETEs (*c*^2^ = 11.10, df = 4, *P*-value = 0.025), specifically that ovary samples tended to have more DETEs with decreased expression during infection (Fig. 5b).

However, similar to our analyses of host sex, there were some unequal associations between pathogen groups and host tissue, suggesting that these patterns may have reflected pathogen-level differences, rather than tissue-level differences. Therefore, we reanalyzed the effect of host tissue in only *Wolbachia* infection datasets, which had datasets of cell culture, ovary, and whole body tissue samples. We found a significant difference in the direction of TE expression between tissues (*c*^2^ = 6.43, df = 2, *P*-value = 0.040).

## Discussion

In this study, we investigated patterns of TE expression during infection in *D. melanogaster*. Other studies have investigated the effect of infection on TE expression in a variety of hosts and conditions, but experimental differences among these studies make it difficult to directly compare their findings and draw broader conclusions about TE dynamics during infection. By bringing together *D. melanogaster* datasets of differing host genotypes, tissue types, sexes, and pathogens, we sought to uncover the influence of these factors on DETEs and identify both unique and broad trends in TE expression during infection.

Of the 184 total DETEs identified in our analyses, 20 were unclassified with an unknown TE class. Though these unknown TEs represented approximately 10% of the DETEs in our study, their unclassified status makes it difficult to comment on their influence on host biology and we have excluded them from the rest of our discussion here. Future studies may reveal more details about these TEs and their role in host gene networks.

### Impact of infection on TE expression

To examine the effect of different infections on TE expression, we examined *D. melanogaster* samples infected with different species of fungal, viral, and bacterial species. Across the different datasets analyzed in this study, infection broadly affected TE expression in flies, though we observed unique differences in the types of TEs and the change in expression depending on the type of pathogen.

First, we analyzed broad trends in whether TEs displayed increased or decreased expression during infection and observed different patterns depending on pathogen group. We observed more TEs with increased expression during infection with fungal pathogens (Fig. 1b). Fungal infections in the tobacco plant (*Nicotiana tabacum*) can cause increased expression of the *Tnt1* retrotransposon (Grandbastien *et al*. 1997). TE insertions can also play a role in gene duplications that are associated with increased resistance to fungal pathogens (Tan *et al*. 2021), suggesting a potential connection between increased TE expression and the host response to fungal infections. However, there is currently not enough information in the literature about how TEs in *D. melanogaster* respond to fungal infection, making it difficult to draw direct comparisons between the TE response in plants during infection to our findings in flies. Nevertheless, our results suggest that fungal infections lead to increased expression of TEs.

We observed the opposite pattern in bacterial infections, where a majority of TEs displayed decreased expression during infection with various bacterial species, including the endosymbiont, *Wolbachia* (Fig. 1). TE insertions are known to affect immune resistance to bacterial pathogens in humans (Bogdan *et al*. 2020) and *D. melanogaster* (Ullastres *et al*. 2021), but it is less clear if TE expression is directly related to bacterial infection resistance. Our results suggest that this may be true, based on our comparison between conventional microbiome and novel infection datasets. We analyzed two datasets comparing flies with and without the conventional gut microbiome and found only one significant DETE associated with conventional microbiome presence across both datasets. In contrast, novel bacterial infections resulted in several DETEs, suggesting that TE expression is significantly changed during infection and may play a role in the bacterial infection response. However, similar to Troha and colleagues (2018) who observed that bacterial pathogenicity did not directly correlate with the number of host genes regulated during infection, we also found that the number of DETEs did not correlate with the severity of bacterial infection.

Following this trend, we observed that *Wolbachia* infections resulted in many DETEs, despite the fact that *Wolbachia* infections are native and non-pathogenic in *D. melanogaster*. Infection with *Wolbachia* was also similar to other bacterial infections by generally decreasing TE expression during infection (Fig. 1d). These results agree with others that have found *Wolbachia* infection decreases TE activity (Touret *et al*. 2014). Another study also showed that *Wolbachia* can differentially affect expression of some TEs in *D. melanogaster*, both increasing and decreasing TE expression depending on TE identity and host genotype (Eugénio *et al*. 2023). While it is plausible that changes in TE expression during infection with other pathogens may be related to host infection responses, this reasoning does not explain why infection with *Wolbachia* should alter TE expression in its host. We discuss more on the effect of *Wolbachia* on its host in the following sections.

These similarities and differences between pathogens, particularly that fungal infections increased TE expression while bacterial infections decreased TE expression, raise interesting questions about whether these patterns are driven by the mechanism of infection for each pathogen. One mechanism to consider is whether each pathogen operates via intracellular or extracellular infections in flies, which are combatted by different immune reactions in *Drosophila* (Kurata 2010). Most fungi are intracellular pathogens in flies, which could be related to the increased TE expression in these infections, while many bacterial infections are extracellular. If TEs are responding as part of the immune system during infection, this could explain why we observed different patterns in TE expression if different immune response networks are activated during different types of infections.

Unfortunately, this correlation breaks down in several ways. First, though *Wolbachia* is an intracellular infection, infections with *Wolbachia* differed from other intracellular fungal infections by decreasing expression of a majority of TEs. *Drosophila* immune responses to fungal pathogens also share more similarities to bacterial pathogens (Lemaitre *et al*. 1997), though we observed opposite patterns in TE expression between fungal and bacterial infections. These interesting patterns in TE expression suggest some divide between bacterial pathogens vs fungal pathogens, though it may not be related to the mode of infection.

Surprisingly, we found very few DETEs during infection with viruses in our datasets (Fig. 1c). This seems to contrast with the well-documented relationship between viruses and TEs, which consistently points to increased TE expression during viral infection (for review, see Hale 2022). There are a couple of mechanisms driving this observed relationship, one of which involves the upregulation of TEs during viral infection as part of the host immune response (Hale 2022). However, increased TE expression can also be a result of viral pathogens disrupting piRNA regulatory pathways, either through oversaturation or by directly manufacturing inhibitors, which can result in de-repression of native TEs (Durdevic *et al.* 2013; van Gestel *et al*. 2014; Roy *et al*. 2020).

However, our results agree with those found by Roy and colleagues (2020), whose samples are included in our analyses here. They found no detectable difference in TE transcript amount in ovary samples, and a very small decrease in TE transcripts during infection in carcass samples. This lack of change in TE expression in ovaries may reflect regulation strategies to protect gametes from TE expression changes (Roy *et al.* 2020). Additionally, both SINV and ZIKV infections are typically non-lethal in *D. melanogaster* (Xu and Cherry 2014; Liu *et al*. 2018), which may also explain why few TEs were differentially expressed in our datasets. Of the few DETEs we identified, we did observe increased expression of the *Doc* TE family, which is known to play a role in virus resistance (Magwire *et al*. 2011; Barrón *et al*. 2014). Future work to compare TE expression in flies infected with lethal and non-lethal viruses may reveal if more lethal infections lead to more drastic TE activation.

### Impact of infection on TE class and superfamily

In addition to the direction of expression, we also analyzed what types of TEs were differentially expressed during infection. Across all infections, most DETEs were RNA retrotransposons, with a small number of DNA transposons differentially expressed in *Wolbachia* infection datasets (Fig. 1). Other studies have found that DNA transposons are the most active TEs in *D. simulans* (Kofler *et al*. 2015), but our results agree with others that have found retrotransposons, specifically LTR elements, to be the most active elements in *D. melanogaster* (Kofler *et al*. 2015).

We also observed little or no RC DNA transposons across all infections, and this was significantly less than expected in bacterial and *Wolbachia* infections. RC elements make up approximately 14% of TEs in the *D. melanogaster* genome, with the RC superfamily *DINE-1* estimated to have the highest copy number of repeats of any TE superfamily (Thomas *et al*. 2014). *DINE-1* elements are often involved in gene duplications, some of which have been linked to insecticide resistance in *Drosophila* (Carareto *et al*. 2014). Yet, despite their prevalence, we observed very few RC elements that were differentially expressed during infection in *D. melanogaster*.

We found that, with the exception of the unclassified TEs, the classes and superfamilies of TEs expressed during fungal infections did not significantly differ from genome proportions, suggesting no preference for the types of TEs affected by fungal infection. However, bacterial infections, including *Wolbachia* infections, affected significantly more TEs from the *Copia* superfamily than expected based on the prevalence of these TEs in the genome. *Copia* elements are known to regulate numerous host genes in *Drosophila* related to development (for review, see Moschetti *et al*. 2020). We observed relatively strong expression (>2 log_2_-fold change) of *Copia* elements across multiple host genotypes and tissue types in *Wolbachia* infection datasets (Supplementary Table 1), suggesting a potential role for these elements during the host response to infection.

Bacterial infections differed significantly from other types of infections and were more likely to affect LINE elements (Fig. 1a) and TEs from the *Jockey* superfamily (Fig. 2a). Of particular note were the transposon families *HeT-A* and *TART*, which decreased in expression during bacterial infection across several datasets. Both of these TE families are known to affect telomere elongation and chromosome stability in flies (Frydrychova *et al*. 2008). Other studies have shown that flies modulate gene expression related to stress and cell homeostasis during bacterial infection (Troha *et al*. 2018), which would include processes relating to telomeres and chromosome structure. Other notable TE families that were differentially expressed during bacterial infection include *invader*, *BURDOCK*, and *BS* elements, which have been linked to expression of immune-related genes that increase infection resistance in *D. melanogaster* (Ullastres *et al*. 2021). Overall, these patterns suggest a decrease in TE activity related to normal cell maintenance processes, perhaps in favor of increasing resources dedicated toward infection resistance.

*Wolbachia* infections differed from infections with other bacterial species, affecting more TEs from the LTR class (Fig. 1d) and the *Ty3* superfamily (Fig. 2d). Other studies have found that *Wolbachia* infection can decrease expression of *Ty3* elements in *D. melanogaster* (Touret *et al*. 2014), but this effect may differ depending on host genotype (Eugénio *et al*. 2023). Though we did not find a significant effect of genotype, we observed that the effect of *Wolbachia* infection can differ across TEs and datasets, with *Wolbachia* infection increasing and decreasing the expression of various *Ty3* elements. This finding may relate to *Wolbachia*'s ability to alter host gene expression that has been observed in other studies (He *et al*. 2019; Frantz *et al*. 2023). TEs from the *Ty3* superfamily have been shown to act as promoters and insulators of host genes (Moschetti *et al*. 2020), suggesting a potential connection between *Wolbachia*'s effect on TE expression also resulting in changes to gene expression in its hosts.

*Wolbachia* infection included the most DNA DETEs of any infection type and affected significantly more TEs from the *Tc1/Mariner* superfamily than expected. This was of particular interest to us because of *Wolbachia*-associated plastic recombination in *D. melanogaster* (Singh 2019; Bryant and Newton 2020; Mostoufi and Singh 2022). DNA transposons are associated with increased recombination rate in the wood white butterfly (*Leptidea sinapis*) (Palahí I Torres *et al*. 2023) and in *C. elegans* (Duret *et al*. 2000). Additionally, heat shock can cause increased gene expression of the *Tc1-mariner* retrotransposon in *C. elegans*, leading to increased DNA double-strand breaks (Kurhanewicz *et al*. 2020). The mechanism behind how *Wolbachia* alters recombination rate in *D. melanogaster* is currently unknown, but these results suggest a potential connection between increased expression of DNA transposons and increased recombination rate that is facilitated by *Wolbachia*. Future experiments which directly test this hypothesis may shed further light on this potential mechanism for *Wolbachia*-associated plastic recombination.

### Impact of host factors on TE expression during infection

We also examined how host factors affected TE expression. We analyzed differences between female and male fly samples, in addition to a cell culture of unknown sex, to determine whether host sex significantly changed TE expression during infection. Sex is known to affect the susceptibility and intensity of infections in humans, with women generally less susceptible to infection due to more robust immune responses compared to men (Klein and Flanagan 2016). *Drosophila* males and females also exhibit several differences in their responses to infection, including sex chromosome-linked variation in immune responses (Taylor and Kimbrell 2007; Hill-Burns and Clark 2009), as well as differences in behavioral responses to infection (for review, see Belmonte *et al.* 2020). However, unlike in humans, there is no one sex in *D. melanogaster* which is overall more responsive to infection, but different types of infections are biased toward male or female survival (Belmonte *et al*. 2020). Additionally, TEs are known to be involved in sexual development and other sex-specific forms of gene expression (for review, see Dechaud *et al*. 2019).

When analyzing for the effect of host sex, we found that differences in TE expression were largely driven by pathogen due to sex biases in several of the datasets. Within fungal infections, which had equal proportions of male and female samples, sex did not significantly impact expression or class proportions of DETEs. However, we still observed that female samples had more DETEs than male samples. These findings suggest that host sex may influence the number of TEs differentially expressed during infection, but not necessarily the types of TEs affected or whether expression is increased or decreased. This finding is particularly interesting in light of other reports which demonstrate that males are more likely to survive fungal infections than females (Taylor and Kimbrell 2007; Shahrestani *et al*. 2018). So, while our results suggest that female flies may see greater changes in TE expression during infection, these changes may not directly correspond to infection resistance to fungal pathogens. The connection between host sex and TE expression and infection resistance may instead play a larger role in other types of infections, particularly with bacterial or viral pathogens. Further investigations of the effect of sex and TE expression during infections with bacterial or viral pathogens would help to clarify this relationship.

In addition to host sex, we also evaluated the effect of host genotype during infection (Fig. 4). We analyzed a total of 16 genotypes and found no significant differences in TE class proportions across host genotypes. One host genotype, *CantonS*, was present in both bacterial and fungal infection datasets, but showed very few similarities within the genotype and was more similar to other fly genotypes within the same pathogen infection group. These results suggest that the influence of host genotypes on TE expression is smaller than the influence of the pathogen.

Host genotype is known to influence TE copy number and insertion location between species and populations (Barrón *et al*. 2014; Signor 2020). Therefore, it was expected that TE copy number and location would vary between the genotypes in our datasets. Indeed, we observed differences in base mean counts of the same TEs across different samples (Supplementary Table 1). TE insertions and copy number can affect TE expression (Lee and Langley 2010), but the piRNA pathway in *Drosophila* also employs copy-dependent silencing of TEs (Kelleher *et al*. 2020). By directly comparing uninfected and infected flies from the same dataset, our analyses controlled for differences in copy number between different genotypes and found that host genotype does not significantly affect the change in TE expression during infection.

Sample tissue also varied across the datasets used in this study, including samples from brain, cell culture, carcass, intestine, ovary, and whole body samples (Fig. 5). Host tissue was associated with significant differences in the direction of TE expression, but differences in TE class and superfamily were nonsignificant. Specifically, ovary tissue samples generally had decreased TE expression, while whole body tissues showed equal proportions of DETEs with increased or decreased expression. Outside of infection, hosts differentially regulate TE expression in different tissues, where somatic expression is generally repressed compared to germline expression (Haig 2016). However, one experiment found the opposite to be true during infection; in Roy *et al*. (2020), TE expression during viral infection was much higher in somatic tissues than in germline tissues, which was confirmed during our analysis of the same data.

Taken together, these results suggest a small role of host factors in influencing TE expression during infection. Although host sex and some tissue samples showed shared patterns in the direction of TE expression, other samples showed little consistency within groups and were more similar to samples from the same pathogen group. In contrast, samples from the same type of infection shared many more patterns in TE expression, suggesting that infection type had a stronger influence on TE expression than host factors. These findings differ from other studies analyzing non-TE gene expression during infection, where host genotype had a larger effect than infection status in *D. melanogaster* (Frantz *et al.* 2023) and the type of pathogen infection in humans (Idaghdour *et al*. 2012). These somewhat contradictory results may relate to differences in the ways that host genes and TEs are activated or suppressed during infection. A majority of TEs are repressed by host mechanisms, which may become overwhelmed during infection and allow for previously silenced TEs to become active. This has been observed during infections in mammals (van Gestel *et al*. 2014) and flies (Roy *et al*. 2020). Therefore, the type of infection may impact which host defense mechanisms are activated and become unable to regulate TE expression.

## Conclusions

TEs can play crucial roles in the host's immune system response to infection as has been demonstrated in humans, mice, flies, and more. However, our understanding of these interactions is limited by differences between pathogen and host factors in each study, affecting our ability to identify broader patterns in TE responses to infection. Our work presented here combines gene expression data from multiple infection studies in *D. melanogaster* to illuminate the influence of pathogen and host factors on TE activity during infection. We find that TE activity is strongly affected by differences in pathogen infection, while the effect of host factors is comparatively smaller. Future experimental work in flies, as well as additional comparative studies in other model organisms, would help to expand our understanding of TE activity during infection even more broadly.

## Supplementary Material

jkae171_Supplementary_Data

## Data Availability

All RNA-seq samples used in these analyses are available online via the associated BioProject records for each individual dataset, which are available in both Table 1 and Supplementary Table 1. The full list of DETEs identified in this study is available in Supplementary Table 2. Results from the generalized linear model are available in Supplementary Table 3; all other results are presented in the paper. All programs used for analyses are described in the Methods section and freely available for installation in Unix/Linux or RStudio environments. Example code and scripts are available on the Singh Lab GitHub page (https://github.com/SinghLab-Oregon/InfectionAndTEs) or through Zenodo (https://zenodo.org/doi/10.5281/zenodo.12752065). Supplemental material available at G3 online.
