# Supplementary material for: Pathogen infection alters the gene expression landscape of transposable elements in Drosophila melanogaster
Source: G3 (Bethesda). 2024 Aug 12;14(9):jkae171. doi: 10.1093/g3journal/jkae171 (PMC11373657; doi:10.1093/g3journal/jkae171)
Supplement: jkae171_Supplementary_Data [file jkae171_supplementary_data.zip › Supplemental_Material_Legends_G3-2024-405230.docx]

**SUPPLEMENTAL MATERIAL CAPTIONS**

**S1 Table. Additional information on datasets used in present study, including: Read Count, Sample Size, number of DETEs, and the Control group.**

**S2 Table. Full list of differentially expressed transposable elements from each dataset.**

**S3 Table. Results from the generalized linear model on TE expression changes.**
